# Social Determinants of Health and Long-Term, Device-Based Sleep Health

**DOI:** 10.1001/jamanetworkopen.2026.43802

**Published:** 2026-11-10

**Authors:** Esha Narla, Fang Zhu, May A. Beydoun, Dayna A. Johnson, Tené T. Lewis, Daniel J. Gottlieb, Tianyi Huang

**Affiliations:** 1 Laboratory of Epidemiology and Population Sciences, Intramural Research Program, National Institute on Aging, Baltimore, Maryland; 2 Department of Epidemiology, Rollins School of Public Health, Emory University, Atlanta, Georgia; 3 Division of Sleep and Circadian Disorders, Departments of Medicine and Neurology, Brigham and Women’s Hospital, Harvard Medical School, Boston, Massachusetts

## Abstract

**Importance:**

Prior research on social determinants of health (SDOH) and sleep health has largely relied on subjective sleep measures, primarily self-reported sleep duration. Evidence remains limited on the associations between SDOH and wearable-derived long-term sleep patterns in large-scale populations.

**Objective:**

To examine how SDOH (overall and by domain) are associated with long-term, wearable-derived sleep duration and variability.

**Design, Setting, and Participants:**

This cross-sectional study included participants from the All of Us Research Program enrolled between 2018 and 2023 who contributed at least 180 days of wearable data, including pre-enrollment historical records, and completed the SDOH survey. Data analysis was conducted from September 2025 to June 2026.

**Exposures:**

Measures of overall and domain-specific SDOH, including social and community context, economic stability, neighborhood environment, and health care access and quality, were derived from validated surveys and standardized to *z* scores.

**Main Outcomes and Measures:**

Mean daily sleep duration (in minutes) and day-to-day sleep duration SD across all available days were calculated from device-estimated main sleep episodes.

**Results:**

Among 12 403 participants (mean [SD] age, 57.8 [15.2] years; 8719 female [70.3%]; 10 946 White [88.3%] and 1457 members of minority racial groups [11.7%]; 756 Hispanic [6.1%]), 9052 individuals (73.0%) had a college degree or above, with a mean (SD) sleep duration of 406.1 (44.9) minutes and a mean (SD) sleep duration SD of 76.9 (20.6) minutes. Participants were monitored for a median (range) of 982 (180 to 4133) days. Compared with participants in the lowest SDOH tertile, those in the highest tertile (indicating more favorable social and structural conditions) had 5.82 minutes (95% CI, 3.84 to 7.80 minutes) longer sleep duration and 4.79 minutes (95% CI, −5.69 to −3.89 minutes) lower sleep duration SD in the fully adjusted model. When examining individual SDOH domains, economic stability showed the strongest associations with mean sleep duration (eg, 10.70 minutes; 95% CI, 5.30 to 16.10 minutes) and sleep duration SD (eg, −8.70 minutes; 95% CI, −11.20 to −6.26 minutes). Limited subgroup heterogeneity in the association was observed across age, sex, race, and ethnicity.

**Conclusions and Relevance:**

In this study, more favorable SDOH conditions were associated with longer and more regular habitual sleep duration over the long term, emphasizing the importance of social and structural factors when evaluating sleep health.

## Key Points

**Question:**

What is the association between social determinants of health (SDOH) and long-term, wearable-derived sleep duration and regularity?

**Findings:**

In this cross-sectional study of 12 403 participants from the All of Us Research Program with valid device-based sleep data, participants with more favorable SDOH across 4 domains had significantly longer mean daily sleep duration and lower day-to-day sleep duration variability.

**Meaning:**

In this study, more favorable SDOH were associated with longer and more regular device-estimated habitual sleep, suggesting that sleep health may be relevant to understanding the association between social disadvantage and health disparities.

## Teaser

This cross-sectional study examines the association between social determinants of health and long-term sleep duration and variability as measured by wearable device.

## Introduction

Sleep health is a multidimensional construct encompassing factors such as duration, regularity, continuity, satisfaction, and architecture, all of which contribute to physical, mental, and neurobehavioral well-being.^1^ Among these, sleep duration and regularity are considered core dimensions and have been consistently associated with adverse cardiometabolic outcomes and increased mortality in prior research.^2,3,4,5,6,7,8,9,10^ In particular, previous studies have suggested a U-shaped association between sleep duration and adverse health outcomes^2,3,4,5,6^ and have reported consistently higher cardiometabolic risk among individuals with irregular sleep pattern.^7,8,9,10^ In the US, poor sleep has become a major public health concern, with persistently high prevalence for insufficient sleep, increasing trends for self-reported sleep problems over time, and marked sleep health disparities across populations.^11,12,13^

Social determinants of health (SDOH), including living conditions and social identities beyond traditional socioeconomic factors, are closely associated with sleep health.^14^ Lower socioeconomic status, often defined by educational attainment and income, has been associated with an increased likelihood of both short and long self-reported sleep duration.^15^ Substantial sleep health disparities have been observed among marginalized populations, such as members of racial and ethnic and sexual minority groups.^16,17^ For example, previous studies suggested that members of racial and ethnic minority groups, such as Black or Mexican American adults, exhibited shorter sleep durations and lower sleep efficiency based on a minimum of 4-day actigraphy measures.^17^ Neighborhood disadvantage has also been associated with worse self-reported sleep health, shorter objective sleep duration, and greater objective sleep irregularity, with stronger associations observed among Black and Hispanic participants and in those living in more deprived areas.^18,19,20^

Despite increasing evidence associating SDOH with sleep health, important limitations remain. Much of the existing research relies on subjective measures, particularly self-reported sleep duration. While wrist actigraphy has been incorporated in more recent studies, these efforts are often constrained by small sample sizes and short monitoring periods.^21,22,23,24,25^ Additionally, most prior studies focused on single domains rather than a comprehensive assessment of SDOH in association with sleep health.^26^ We aimed to address these limitations by integrating long-term, wearable-derived sleep data and comprehensive SDOH assessment in the All of Us Research Program. We hypothesized that poorer SDOH, including lower economic stability, reduced health care access and quality, and adverse social and neighborhood environments, would be associated with worse sleep health, characterized by wearable-derived shorter and more variable sleep duration.

## Methods

### Study Population

Data for this cross-sectional study were obtained from the National Institutes of Health All of Us Research Program, an ongoing nationwide longitudinal research initiative in the US (initiated in May 2018) designed to enroll more than 1 million participants and create a diverse cohort. The All of Us Controlled Tier Curated Data Repository version 8, released in February 2025, contains data from more than 633 000 participants enrolled through October 1, 2023, integrating survey responses, electronic health records, physical measurements, and wearable device data.

The All of Us Research Program protocol and materials were reviewed and approved by the All of Us Institutional Review Board, and participants provided informed consent for participation and research use of their data. This secondary analysis was approved by the National Institutes of Health Institutional Review Board and is reported following the Strengthening the Reporting of Observational Studies in Epidemiology (STROBE) reporting guideline.^27^

A total of 44 940 participants were initially identified based on completion of the SDOH survey and contribution of wearable data (eFigure 1 in Supplement 1). To minimize the influence of extreme or potentially implausible values, we restricted analyses to daily main sleep episodes lasting 180 to 840 minutes. Furthermore, to ensure reliable estimation of habitual sleep patterns and reduce sensitivity to short-term disruptions or atypical periods, we excluded participants with fewer than 180 days of valid wearable sleep data, leaving 14 960 eligible individuals. This threshold was selected a priori as a pragmatic balance between longitudinal coverage and sample retention. Additional exclusions were applied based on missing SDOH survey items, yielding a final analytic sample of 12 403 participants.

### Assessment of SDOH Exposures

SDOH exposures were assessed using the All of Us SDOH survey instruments, which capture participants’ perceptions of their social, economic, and physical environments. The surveys comprise 16 constructs spanning 4 SDOH domains: (1) social and community context, (2) economic stability, (3) neighborhood environment, and (4) health care access and quality.^28^ For example, the social and community context domain includes constructs such as social cohesion, social support, loneliness, perceived discrimination, perceived stress, daily spiritual experiences, religious service attendance, and English proficiency, each assessed via validated survey instruments.

Responses were numerically coded according to All of Us guidelines.^28^ Item responses were summed within each construct to generate raw scores, which were minimum-maximum normalized to a 0 to 1 scale, with higher values reflecting more favorable conditions. Rescaled construct scores were combined into domain-level composite scores and summed to derive an overall SDOH score. Overall and domain-specific scores were *z* standardized to facilitate comparison and prevent domains with more constructs from disproportionately influencing the analyses. Most survey instruments showed strong internal consistency (Cronbach α >0.80).^28^ Details on the instruments and scoring procedures are provided in eTable 1 in Supplement 1. Modest correlations among the 4 domains (Pearson correlation <0.28) suggested that they represented distinct aspects of SDOH (eFigure 2 in Supplement 1).

### Assessment of Wearable-Derived Sleep Measures

Device-estimated sleep measures were obtained from the wearable device (Fitbit, Fitbit, Inc) through the Bring Your Own Device (BYOD) and Wearables Enhancing All of Us Research (WEAR) substudies. These initiatives allow participants to contribute wearable data through personally owned devices or devices provided by the program.^29^ As of October 1, 2023, All of Us version 8 included more than 59 000 participants who had contributed wearable device data. Given the study design, Fitbit Charge or Versa models were used in WEAR participants, whereas 41 models were documented in BYOD participants.^30^ Furthermore, BYOD participants (approximately 70% of the sample) contributed historical wearable data that predated their enrollment and SDOH assessment, in addition to postenrollment wearable records.

Sleep health was operationalized using 2 primary measures: mean sleep duration and sleep duration regularity, defined as the SD of nightly sleep duration across the observation period (eFigure 3 in Supplement 1). The 2 sleep measures were weakly correlated (Pearson correlation = −0.04). Of note, leveraging participants’ own devices is a common feature of naturalistic longitudinal wearable research that facilitates assessment of habitual sleep over extended periods and addresses key limitations of prior studies based on short-term actigraphy or self-reported sleep. Although prior validation studies supported broadly comparable group-level estimates of sleep duration across several newer wearable device models,^31,32^ variation in device hardware and proprietary processing over time may introduce residual measurement error.

### Covariate Assessment

Covariates were selected based on prior literature and conceptual relevance. Demographic covariates included self-reported measures: age (continuous), sex assigned at birth (female, male), race (American Indian or Alaska Native; Asian; Black, African American, or African; Middle Eastern or North African; Native Hawaiian or Other Pacific Islander; White; and more than 1 racial group, with groups other than White combined as *racial minority group* because some individual groups were too small to report separately under the All of Us Data and Statistics Dissemination Policy), and ethnicity (Hispanic, not Hispanic). Socioeconomic covariates included educational attainment (high school or less, some college, college graduate, advanced degree), annual household income (<$35 000, $35 000-$74 999, $75 000-$99 999, $100 000-$149 999, $150 000-$199 999, >$200 000, missing), and employment status (employed, not employed, retired). While these sociodemographic factors are related to SDOH, they do not fully capture the broader social, environmental, and structural conditions assessed by the SDOH survey. We further considered 2 behavioral covariates: smoking and alcohol use. All covariates previously described were collected at enrollment. Depressive symptoms were assessed using the 9-item Patient Health Questionnaire (PHQ-9) among 4673 participants of our sample who completed the COVID-19 Participant Experience survey.^33^

### Statistical Analysis

Linear regression models were used to examine associations between SDOH exposures (overall and individual domain scores) and continuous sleep measures (mean sleep duration and sleep duration SD). Model diagnostics supported the linear regression assumptions; no transformation was applied. Overall and domain scores were modeled in tertiles and as a continuous variable, with an exception for economic stability, which was examined in nominal categories due to the limited score variability resulting from fewer questions. We considered several sequentially adjusted multivariable models: Model 1 adjusted for demographic factors, while model 2 additionally adjusted for socioeconomic factors, model 3 further adjusted for behavioral factors, and model 4 included mutual adjustment for all SDOH domains (for domain-specific analyses). As a sensitivity analysis, we further adjusted for depressive symptoms among participants with available PHQ-9 data.

Given that many prior studies reported U-shaped findings for sleep duration,^2,3,4,6^ we conducted secondary analyses by categorizing sleep duration into short, intermediate, and long groups based on its sample distribution (<6, 6-8, and >8 hours, respectively). Multinomial logistic regression was used to evaluate associations between SDOH exposures and sleep duration categories, adjusted for covariates in model 3. Prespecified stratified analyses were conducted by age, sex, race, and ethnicity to explore potential heterogeneity of subgroup associations, assessing statistical significance of the interaction and subgroup-specific effect estimates. All statistical analyses were conducted in the All of Us Researcher Workbench using RStudio version 2025.09.0 (RStudio) in R statistical software version 4.5.3 (R Project for Statistical Computing). All tests were 2 sided, with *P* < .05 considered statistically significant. Data analysis was conducted from September 2025 to June 2026.

## Results

Among 12 403 participants (mean [SD] age, 57.8 [15.2] years; 8719 female [70.3%]; 10 946 White [88.3%] and 1457 members of minority racial groups [11.7%]; 756 Hispanic [6.1%]), 9052 participants (73.0%) had a college degree or above, 7897 (64.4%) were employed, and 5593 (45.2%) had annual household incomes above $100 000 vs 1348 (10.9%) below $35 000 (Table 1). Participants with higher SDOH *z* scores were more likely to be White, male, older, retired, and have higher income and education and lower depressive symptoms and were less likely to be unemployed or current smokers. Over a mean (SD) monitoring period of 1156 (777) days (median [range], 982 [180-4133] days) and a total of 14 337 868 daily records, participants had an overall mean (SD) sleep duration of 406.1 (44.9) minutes and sleep duration SD of 76.9 (20.6) minutes. Participants with higher SDOH *z* score had longer mean sleep durations and less sleep irregularity. Compared with the overall All of Us study population of 633 546 individuals, participants in our study were more likely to be female, White, non-Hispanic, and employed and to have higher education and income (eTable 2 in Supplement 1).

**Table 1.** Study Sample Characteristics

Characteristic	Participants, No. (%)			
	Overall (N = 12 403)	Tertile 1 (n = 4135)	Tertile 2 (n = 4134)	Tertile 3 (n = 4134)
Age at enrollment, mean (SD), y	53.2 (15.2)	48.7 (15.2)	53.7 (15.2)	57.3 (14.0)
Sex				
Female	8719 (70.3)	3180 (76.9)	2914 (70.5)	2625 (63.5)
Male	3684 (29.7)	955 (23.1)	1220 (29.5)	1509 (36.5)
Race				
White	10 946 (88.3)	3577 (86.5)	3681 (89.0)	3688 (89.2)
Racial minority group^a^	1457 (11.7)	558 (13.5)	453 (11.0)	446 (10.8)
Hispanic	756 (6.1)	281 (6.8)	219 (5.3)	256 (6.2)
Employment status				
Employed	7987 (64.4)	2775 (67.1)	2687 (65.0)	2525 (61.1)
Not employed^b^	1417 (11.4)	740 (17.9)	389 (9.4)	288 (7.0)
Retired	2999 (24.2)	620 (15.0)	1058 (25.6)	1321 (32.0)
Annual income, US $				
<35 000	1348 (10.9)	777 (18.8)	368 (8.9)	203 (4.9)
35 000-74 999	2948 (23.8)	1220 (29.5)	922 (22.3)	806 (19.5)
75 000-99 999	1877 (15.1)	562 (13.6)	683 (16.5)	632 (15.3)
100 000-149 999	2711 (21.9)	745 (18.0)	940 (22.7)	1026 (24.8)
150 000-199 999	1286 (10.4)	302 (7.3)	480 (11.6)	504 (12.2)
>200 000	1596 (12.9)	355 (8.6)	530 (12.8)	711 (17.2)
Missing	637 (5.1)	174 (4.2)	211 (5.1)	252 (6.1)
Education				
High school or less	747 (6.0)	310 (7.5)	211 (5.1)	226 (5.5)
Some college	2604 (21.0)	1050 (25.4)	823 (19.9)	731 (17.7)
College graduate	4128 (33.3)	1356 (32.8)	1401 (33.9)	1371 (33.2)
Advanced degree	4924 (39.7)	1419 (34.3)	1699 (41.1)	1806 (43.7)
Depressive symptom score, mean (SD)^c^	6.8 (5.6)	9.3 (6.3)	6.3 (4.9)	4.5 (4.3)
Mean sleep duration, mean (SD), min	406.1 (44.9)	404.5 (46.0)	406.5 (44.6)	407.4 (44.0)
Sleep duration SD, mean (SD), min	76.9 (20.6)	81.5 (22.1)	76.0 (19.8)	73.2 (19.0)
No. of days with sleep measurements, mean (SD)	1156 (778)	1064 (731)	1161 (779)	1243 (811)
Current smoker	464 (3.7)	252 (6.1)	129 (3.1)	83 (2.0)
Current drinker	12 077 (97.4)	4011 (97.0)	4031 (97.5)	4035 (97.6)
SDOH domains (*z* scored), mean (SD)				
Social and community context	0 (1)	−0.62 (0.92)	−0.10 (0.76)	0.72 (0.82)
Economic stability	0 (1)	−0.81 (1.38)	0.37 (0.35)	0.44 (0.07)
Neighborhood environment	0 (1)	−0.61 (1.08)	−0.02 (0.83)	0.63 (0.61)
Health care access and quality	0 (1)	−0.76 (1.15)	0.09 (0.69)	0.67 (0.40)
Overall SDOH score	0 (1)	−1.14 (0.80)	0.19 (0.22)	0.95 (0.29)

^a^ Includes American Indian or Alaska Native; Asian; Black, African American, or African; Middle Eastern or North African; Native Hawaiian or Other Pacific Islander; and more than 1 racial group.

^b^ Includes individuals who were unemployed, homemakers, students, or those unable to work.

^c^ Depressive symptoms were assessed by the 9-item Patient Health Questionnaire among 4673 individuals who participated in the COVID-19 Participant Experience survey.

### Associations Between SDOH and Mean Sleep Duration

Higher overall SDOH scores were associated with longer mean sleep duration across all models (Table 2). After adjusting for demographic factors, compared with participants in the lowest tertile of SDOH *z* scores, mean sleep duration was 4.55 minutes (95% CI, 2.65-6.46 minutes) higher in the middle tertile and 7.80 minutes (95% CI, 5.85-9.74 minutes) higher in the highest tertile. Each 1-unit increment in the SDOH *z* score was associated with 3.67 minutes (95% CI, 2.87-4.46 minutes) longer mean sleep duration (*P *for trend < .001). Results were slightly attenuated but remained similar after additional adjustment for sociodemographic and lifestyle factors (β = 2.78 minutes; 95% CI, 1.95-3.60 minutes; *P *for trend < .001). In model 3, the highest tertile had 5.82 minutes (95% CI, 3.84-7.80 minutes) higher mean sleep duration than the lowest tertile. A sensitivity analysis with additional adjustment of depressive symptoms (eTable 3 in Supplement 1) showed consistent linear associations.

**Table 2.** Associations Between SDOH and Mean Sleep Duration

SDOH	β (95% CI)			
	Model 1^a^	Model 2^b^	Model 3^c^	Model 4^d^
Overall SDOH score				
Tertile1	0 [Reference]	0 [Reference]	0 [Reference]	NA
Tertile 2	4.55 (2.65 to 6.46)	3.34 (1.42 to 5.26)	3.16 (1.24 to 5.08)	NA
Tertile 3	7.80 (5.85 to 9.74)	6.05 (4.07 to 8.03)	5.82 (3.84 to 7.80)	NA
Per 1-unit increase	3.67 (2.87 to 4.46)	2.90 (2.07 to 3.72)	2.78 (1.95 to 3.60)	NA
*P* for trend^e^	<.001	<.001	<.001	NA
Social and community context				
Tertile 1	0 [Reference]	0 [Reference]	0 [Reference]	0 [Reference]
Tertile 2	4.25 (2.34 to 6.15)	3.21 (1.30 to 5.12)	3.04 (1.13 to 4.95)	1.91 (−0.03 to 3.84)
Tertile 3	3.01 (1.07 to 4.94)	1.95 (0.01 to 3.89)	1.92 (−0.03 to 3.86)	0.30 (−1.71 to 2.31)
Per 1-unit increase	1.32 (0.53 to 2.11)	0.83 (0.03 to 1.62)	0.83 (0.02 to 1.63)	0.09 (−0.74 to 0.93)
*P* for trend	.001	.04	.04	.83
Economic stability				
Food insecure and housing issues	0 [Reference]	0 [Reference]	0 [Reference]	0 [Reference]
Food insecure or housing issues	7.46 (1.91 to 13.00)	6.17 (0.62 to 11.70)	5.15 (−0.40 to 10.70)	4.62 (−0.93 to 10.20)
Food secure and no housing issues	16.00 (10.70 to 21.30)	13.00 (7.66 to 18.40)	11.80 (6.46 to 17.20)	10.70 (5.30 to 16.10)
Per 1-unit increase	2.91 (2.13 to 3.70)	2.31 (1.51 to 3.10)	2.18 (1.39 to 2.97)	1.89 (1.08 to 2.69)
*P* for trend	<.001	<.001	<.001	<.001
Neighborhood environment				
Tertile 1	0 [Reference]	0 [Reference]	0 [Reference]	0 [Reference]
Tertile 2	3.16 (1.27 to 5.06)	2.59 (0.70 to 4.48)	2.42 (0.53 to 4.31)	1.85 (−0.05 to 3.74)
Tertile 3	3.81 (1.90 to 5.71)	2.74 (0.82 to 4.66)	2.49 (0.58 to 4.41)	1.49 (−0.45 to 3.43)
Per 1-unit increase	1.65 (0.87 to 2.43)	1.20 (0.41 to 1.98)	1.10 (0.32 to 1.89)	0.72 (−0.08 to 1.52)
*P* for trend	<.001	.003	.006	.08
Health care access and quality				
Tertile 1	0 [Reference]	0 [Reference]	0 [Reference]	0 [Reference]
Tertile 2	3.95 (2.15 to 5.75)	2.96 (1.16 to 4.76)	2.86 (1.06 to 4.65)	2.13 (0.31 to 3.94)
Tertile 3	5.34 (3.34 to 7.34)	4.23 (2.23 to 6.23)	4.13 (2.13 to 6.13)	3.10 (1.05 to 5.16)
Per 1-unit increase	2.51 (1.73 to 3.30)	1.96 (1.17 to 2.75)	1.90 (1.12 to 2.69)	1.57 (0.75 to 2.38)
*P* for trend	<.001	<.001	<.001	<.001

Abbreviations: NA, not applicable; SDOH, social determinants of health.

^a^ Adjusted for demographic factors (age, sex, race, and ethnicity).

^b^ Model 1 and socioeconomic status (education, income, and employment status).

^c^ Model 2 and behavioral factors (smoking and alcohol use).

^d^ Model 3 and mutually adjusted for individual SDOH domains.

^e^ *P* values for linear trends were calculated by modeling the SDOH score as a continuous variable.

Among individual SDOH domains, economic stability demonstrated a strong positive association with sleep duration after full adjustment (Table 2). Participants who were food secure without housing issues had a 10.70 minutes (95% CI, 5.30-16.10 minutes) longer sleep duration compared with those who experienced both food and housing insecurity (*P* for trend < .001). Better health care access and quality was also associated with longer mean sleep duration after full adjustment. Weaker positive associations were observed for social and community context and neighborhood environment. These associations were attenuated and no longer present after mutual adjustment for other domains of SDOH.

### Associations Between SDOH and Sleep Duration SD

Higher overall SDOH scores were associated with lower sleep irregularity, indicating more consistent day-to-day sleep duration (Table 3). Compared with participants in the lowest tertile of overall SDOH *z* scores, those in the middle and highest tertile showed 4.75 minutes (95% CI, −5.63 to −3.87 minutes) and 7.03 minutes (95% CI, −7.93 to −6.13 minutes) lower sleep duration SD, respectively, in model 1. Every 1-unit increase in the SDOH *z* score was associated with 3.63 minutes (95% CI, −3.99 to −3.26 minutes) lower sleep duration SD (*P* for trend < .001). After further adjustment for socioeconomic and behavioral factors, we found similar results. Participants in the highest tertile had 4.79 minutes (95% CI, −5.69 to −3.89 minutes) lower sleep duration SD compared with those in the lowest tertile in model 3. Additional adjustment for depressive symptoms attenuated the association, although a modest association remained (eTable 3 in Supplement 1).

**Table 3.** Associations Between SDOH and Sleep Duration SD

SDOH	β (95% CI)			
	Model 1^a^	Model 2^b^	Model 3^c^	Model 4^d^
Overall SDOH score				
Tertile 1	0 [Reference]	0 [Reference]	0 [Reference]	NA
Tertile 2	−4.75 (−5.63 to −3.87)	−3.17 (−4.04 to −2.30)	−3.07 (−3.94 to −2.20)	NA
Tertile 3	−7.03 (−7.93 to −6.13)	−4.93 (−5.83 to −4.03)	−4.79 (−5.69 to −3.89)	NA
Per 1-unit increase	−3.63 (−3.99 to −3.26)	−2.56 (−2.93 to −2.19)	−2.49 (−2.87 to −2.12)	NA
*P* for trend^e^	<.001	<.001	<.001	NA
Social and community context				
Tertile 1	0 [Reference]	0 [Reference]	0 [Reference]	0 [Reference]
Tertile 2	−3.98 (−4.86 to −3.09)	−2.57 (−3.44 to −1.71)	−2.48 (−3.35 to −1.61)	−1.70 (−2.58 to −0.82)
Tertile 3	−4.79 (−5.68 to −3.89)	−3.49 (−4.37 to −2.61)	−3.37 (−4.26 to −2.49)	−2.32 (−3.23 to −1.41)
Per 1-unit increase	−2.43 (−2.80 to −2.06)	−1.83 (−2.19 to −1.47)	−1.79 (−2.15 to −1.43)	−1.31 (−1.69 to −0.94)
*P* for trend	<.001	<.001	<.001	<.001
Economic stability				
Food insecure and housing issues	0 [Reference]	0 [Reference]	0 [Reference]	0 [Reference]
Food insecure or housing issues	−9.12 (−11.70 to −6.55)	−6.30 (−8.81 to −3.78)	−5.98 (−8.50 to −3.46)	−5.49 (−8.00 to −2.98)
Food secure and no housing issues	−15.20 (−17.70 to −12.80)	−10.30 (−12.70 to −7.82)	−9.84 (−12.30 to −7.40)	−8.70 (−11.20 to −6.26)
Per 1-unit increase	−2.34 (−2.71 to −1.98)	−1.50 (−1.86 to −1.14)	−1.45 (−1.81 to −1.09)	−1.10 (−1.46 to −0.74)
*P* for trend	<.001	<.001	<.001	<.001
Neighborhood environment				
Tertile 1	0 [Reference]	0 [Reference]	0 [Reference]	0 [Reference]
Tertile 2	−2.11 (−2.99 to −1.23)	−1.22 (−2.08 to −0.36)	−1.15 (−2.01 to −0.29)	−0.62 (−1.48 to 0.24)
Tertile 3	−3.77 (−4.65 to −2.88)	−2.22 (−3.10 to −1.35)	−2.14 (−3.01 to −1.27)	−1.25 (−2.13 to −0.37)
Per 1-unit increase	−1.68 (−2.04 to −1.32)	−1.01 (−1.37 to −0.65)	−0.98 (−1.33 to −0.62)	−0.53 (−0.90 to −0.17)
*P* for trend	<.001	<.001	<.001	.004
Health care access and quality				
Tertile 1	0 [Reference]	0 [Reference]	0 [Reference]	0 [Reference]
Tertile 2	−3.94 (−4.78 to −3.11)	−2.88 (−3.69 to −2.06)	−2.84 (−3.65 to −2.02)	−2.14 (−2.96 to −1.32)
Tertile 3	−4.25 (−5.18 to −3.33)	−3.14 (−4.05 to −2.24)	−3.07 (−3.97 to −2.16)	−1.97 (−2.90 to −1.04)
Per 1-unit increase	−2.25 (−2.61 to −1.89)	−1.57 (−1.93 to −1.22)	−1.54 (−1.90 to −1.18)	−1.03 (−1.40 to −0.66)
*P* for trend	<.001	<.001	<.001	<.001

^a^ Adjusted for demographic factors (age, sex, race, and ethnicity).

^b^ Model 1 and socioeconomic status (education, income, and employment status).

^c^ Model 2 and behavioral factors (smoking and alcohol use).

^d^ Model 3 and mutually adjusted for all domains.

^e^ *P* values for linear trends were calculated by modeling the SDOH score as a continuous variable.

Among individual SDOH domains, strong inverse associations with sleep duration SD were again observed for economic stability across all models (Table 3). Compared with participants experiencing both housing and food insecurity, those without such constraints experienced 8.70 minutes (95% CI, −11.20 to −6.26 minutes) less variability in sleep duration in the fully adjusted model (*P* for trend < .001). Social and community context and health care access and quality showed similar inverse associations. Neighborhood environment showed weaker associations with sleep duration irregularity compared with other SDOH domains, although these associations remained across all models.

### Associations of SDOH With Short and Long Sleep Duration

In further analyses exploring associations with categorical sleep duration, we grouped participants into short (<6 hours; 1832 participants [14.2%]), intermediate (6-8 hours; 10 053 participants [81.1%]), and long (>8 hours; 518 participants [4.2%]) sleep duration. After covariate adjustment, higher overall SDOH score was associated with lower odds of short vs intermediate sleep duration (odds ratio, 0.85; 95% CI, 0.81-0.90), with no association observed for long sleep (Table 4). Inverse associations between SDOH and short sleep duration were consistently observed across individual SDOH domains, whereas only economic stability showed a positive association for long sleep duration (odds ratio, 1.12; 95% CI, 1.01-1.24).

**Table 4.** Associations of SDOH With Short and Long Sleep Duration

SDOH	OR (95% CI)^a^		
	Short (<6 h) (n = 1832)	Medium (6-8 h) (n = 10 053)	Long (>8 h) (n = 518)
Overall SDOH	0.85 (0.81-0.90)	1 [Reference]	1.05 (0.96-1.16)
Social and community context	0.93 (0.89-0.99)	1 [Reference]	0.99 (0.90-1.08)
Economic stability	0.92 (0.87-0.97)	1 [Reference]	1.12 (1.01-1.24)
Neighborhood environment	0.93 (0.88-0.98)	1 [Reference]	1.00 (0.91-1.10)
Health care access and quality	0.89 (0.85-0.94)	1 [Reference]	0.99 (0.90-1.09)

Abbreviations: OR, odds ratio; SDOH, social determinants of health.

^a^ Values are from multinomial logistic regression, with overall and domain-specific SDOH scores modeled as a continuous exposure (per 1-unit increase) and medium sleep duration (6-8 hours) modeled as the reference outcome category.

### Subgroup Associations

In stratified analyses, associations were generally consistent across age (eTable 4 in Supplement 1), sex (eTable 5 in Supplement 1), race (eTable 6 in Supplement 1), and ethnicity (eTable 7 in Supplement 1), with a few statistically significant subgroup differences. We observed a stronger association between overall SDOH and mean sleep duration among participants aged 60 years or older vs younger than 60 years (*P* for interaction = .046) (Figure, A). Such subgroup differences by age were primarily attributed to social and community context (*P* for interaction = .01) and economic stability (*P* for interaction = .07). By contrast, the inverse association between health care access and quality and sleep duration SD was stronger in younger vs older participants (*P* for interaction = .004) (Figure, B). We also observed racial differences in the association between neighborhood environment and mean sleep duration (*P* for interaction = .04) (Figure, C). Among participants in racial minority groups, better neighborhood environment was associated with 2.37 minutes (95% CI, 0.30-4.44 minutes) higher mean sleep duration, and no association was observed in White participants. No appreciable differences were observed for the remaining subgroup stratifications.

**Figure.**
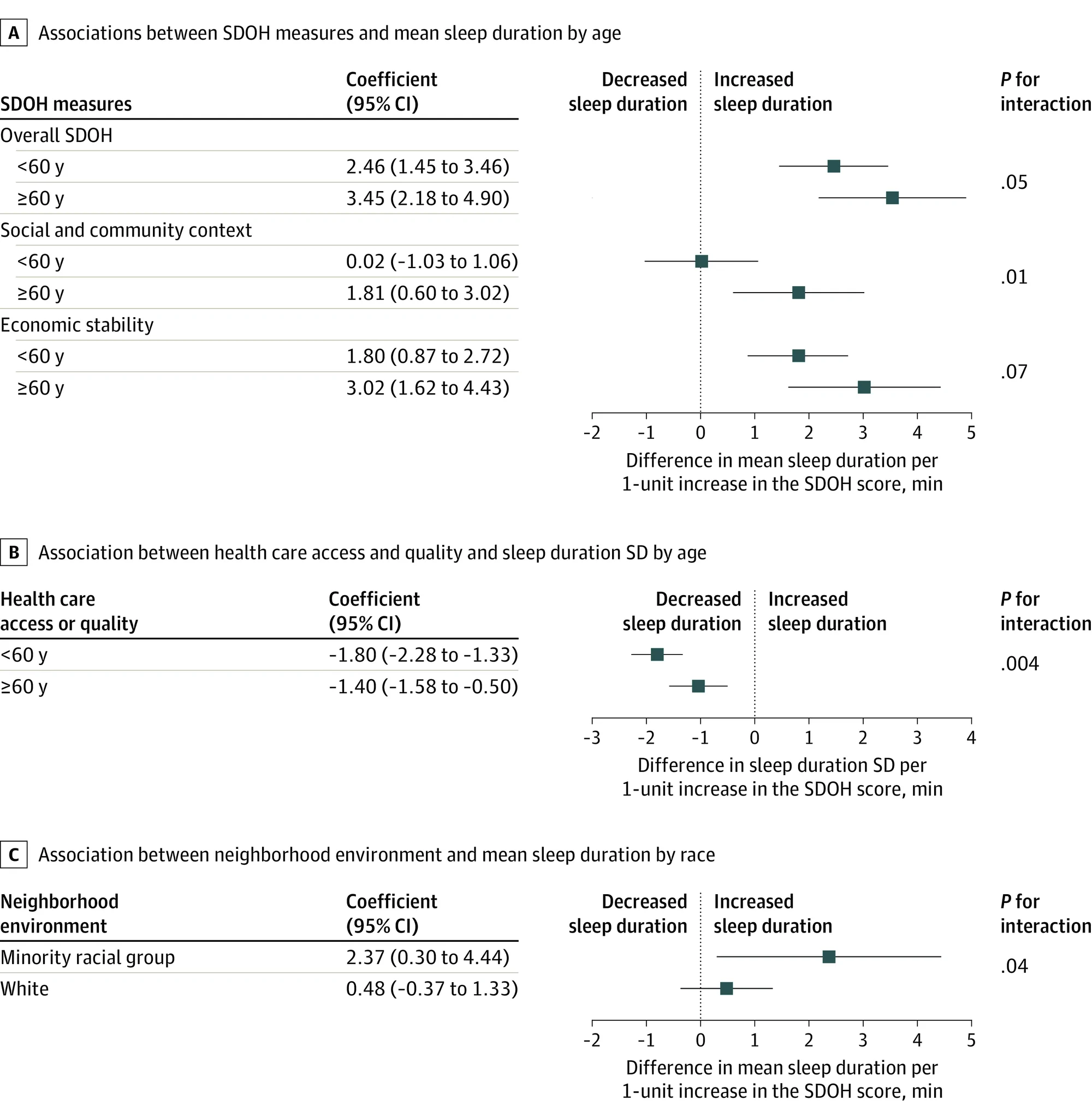
Dot Plots of Selected Subgroup Associations Between Social Determinants of Health (SDOH) and Device-Based Sleep Duration and Regularity Points indicate β coefficients; whiskers, 95% CIs. All estimates were adjusted for age, sex, race, ethnicity, education, income, employment status, smoking, and alcohol use. *P* values for interactions were calculated by the Wald test of the cross-product terms.

## Discussion

Overall, findings from this cross-sectional study support the hypothesis that poorer SDOH conditions are associated with less favorable device-estimated, long-term sleep duration and regularity and corroborate studies using self-reported sleep measures. Better overall and domain-specific SDOH were associated with longer and more regular sleep duration. Notably, associations between SDOH and sleep duration irregularity were generally stronger and more consistent across SDOH domains than those observed for mean sleep duration. Although limited effect modification was observed overall, several differences by age and race were noted, suggesting that these subgroup patterns may warrant further investigation.

Previous studies have predominantly focused on socioeconomic factors, such as education, income, and employment, which have been associated with both short and long self-reported sleep duration.^15,34^ Our study extended beyond individual-level socioeconomic factors and self-reported sleep duration by examining a comprehensive measure of SDOH in association with long-term, wearable-derived mean sleep duration. Within the range of sleep durations well represented in our sample, we observed an approximately linear association between SDOH and mean sleep duration. Although the categorical analyses did not show a clear U-shaped pattern, 4.2% of participants had a mean sleep duration greater than 8 hours, resulting in limited statistical power to detect potential nonlinearity at the upper end of the sleep duration distribution. Therefore, this finding should not be interpreted as evidence that device-estimated sleep duration lacks the U-shaped association reported in studies using self-reported sleep. Furthermore, it should be noted that the conventional cutoffs for defining long sleep were primarily derived from studies using self-reported sleep.^35^ Differences between self-reported and wearable-derived sleep may also be relevant given that prior studies have demonstrated only modest agreement between these measures.^36,37^ Additionally, self-reported long sleep duration may reflect underlying health conditions, such as obesity or depression, rather than true physiological sleep need, potentially contributing to discrepancies between self-reported and objective sleep duration.^38^ Future studies with greater representation of long sleepers are needed to determine whether a U-shaped association is present for wearable-derived sleep and whether established thresholds based largely on self-reported sleep duration are appropriate for objective measurements.

While, to our knowledge, no prior studies have comprehensively examined SDOH in association with wearable-derived sleep duration, our findings are consistent with existing evidence that focused on individual SDOH domains.^26^ For example, neighborhood characteristics, such as social cohesion, perceived safety, and better walkability, have been associated with longer device-estimated sleep duration, and the observed associations appeared to differ by neighborhood.^20,39^ Economic instability, including food insecurity and lower socioeconomic status, has been associated with insufficient sleep duration.^34^ By contrast, our findings that were built on a comprehensive multidomain SDOH framework using long-term, device-estimated sleep measures demonstrated consistent favorable associations with mean sleep duration across all SDOH domains. Notably, prior research suggests that neighborhood conditions may be differentially associated with sleep duration across racial groups,^22^ which is supported by our findings of stronger associations between neighborhood environment and sleep duration among members of racial minority groups. Although the adjusted mean difference in sleep duration comparing the top vs bottom SDOH tertiles was statistically significant (5.82 minutes per day), the clinical relevance of a mean nightly difference of this magnitude over extended periods remains uncertain and requires further investigation. Given that sleep duration is socially patterned, our findings underscore the importance of SDOH, such as housing conditions, economic stability, and neighborhood environment, as correlates of sleep health and key considerations for future interventions.

Limited studies have examined the association between multidomain SDOH and sleep regularity. To our knowledge, only 1 study of 5045 adolescents reported that greater neighborhood disadvantage was associated with higher day-to-day sleep duration irregularity over 21 days.^19^ Our study provided robust evidence that greater sleep irregularity consistently occurred among individuals with poorer SDOH across different SDOH domains. Sleep irregularity has emerged as a potential risk factor associated with cardiometabolic disease, likely through mechanisms related to circadian misalignment.^40^ Rotating night shift work has also been associated with increased cardiovascular risk, further supporting the role of disrupted circadian rhythms in disease development.^41^ Importantly, nonstandard and shift work schedules, which are key drivers of high day-to-day sleep variability,^42^ are more common among individuals with lower socioeconomic status and poorer SDOH, suggesting a plausible explanation for the greater sleep irregularity observed among these groups.^43^ More broadly, disadvantaged social, economic, and neighborhood conditions may be associated with shorter and more irregular sleep duration, potentially reflecting co-occurring psychosocial distress (eg, anxiety and depression)^44^ and environmental disruptors (eg, nighttime noise, air pollution, and light at night).^45,46^ In light of increasing evidence associating sleep irregularity with chronic disease risk,^7,8,9,10^ our findings suggest that sleep irregularity may be relevant to understanding associations between social disadvantage and adverse health outcomes.

### Limitations

This study has several limitations. First, wearable-derived measurements may contain error because the All of Us wearable device dataset includes multiple device models with evolving sleep-estimating algorithms. Second, the sample was mostly White with higher socioeconomic status, limiting generalizability to the broader All of Us cohort or general population. Long-term wearable contributors may differ systematically from noncontributors, potentially introducing healthy volunteer bias common in biobanks.^47^ Associations may be stronger in more diverse samples with wider distributions of SDOH. Third, self-reported SDOH measures may be subject to perception-based biases. Fourth, we examined only 2 sleep characteristics and focused on the main sleep period (excluding compensatory sleep, such as naps), which did not capture the full multidimensional nature of sleep health. Future studies leveraging daily sleep measures could use mixed-effects, location-scale models to characterize longitudinal changes in both mean sleep duration and within-person variability. Furthermore, because commonly used thresholds for short and long sleep are largely informed by studies using self-reported sleep duration, approaches such as quantile regression could examine associations across the full distribution of device-based sleep without imposing prespecified categories, assess the appropriateness of existing thresholds, and potentially clarify the absence of a U-shaped association in our analyses. Fifth, the cross-sectional design precludes causal inference and does not allow us to rule out reverse causation (eg, preexisting poor health may influence both SDOH factors and sleep health). Importantly, the wearable measurement windows were not anchored to SDOH survey completion; thus, sleep observations could precede and/or follow the survey, precluding conclusions about temporal direction. Sixth, residual confounding may remain despite adjustment for key covariates and other SDOH domains.

## Conclusions

In this cross-sectional study of All of Us participants, more favorable SDOH were associated with longer and more regular sleep. This large-cohort cross-sectional study combined long-term, wearable-derived sleep measures with multidomain SDOH, representing one of the first comprehensive assessments of their association. These findings highlight the importance of sleep as a multidimensional construct associated with social and structural determinants. Our study found that sleep duration and regularity varied across SDOH groups, with potential implications for chronic disease disparities. Future work should expand these analyses to include additional sleep dimensions and more diverse populations, including rural communities. Addressing SDOH may represent an important strategy for improving sleep health and reducing health disparities.

## Notes

Supplement 1. eTable 1. Assessment of social determinants of health (SDOH) in the All of Us Research Program eTable 2. Comparison of key sociodemographic characteristics among the overall All of Us Research Program population (version 8), social determinants of health survey respondents, and the current study sample eTable 3. Sensitivity analysis of associations between social determinants of health (SDOH) and the mean and SD of sleep duration among a subset of All of Us participants with depressive symptom assessments eTable 4. Associations of social determinants of health with mean sleep duration and sleep duration SD stratified by age eTable 5. Associations of social determinants of health with mean sleep duration and sleep duration SD stratified by sex eTable 6. Associations of social determinants of health with mean sleep duration and sleep duration SD stratified by race eTable 7. Associations of social determinants of health with mean sleep duration and sleep duration SD stratified by ethnicity eFigure 1. Flowchart of the analytical sample eFigure 2. Correlation between overall SDOH and SDOH domains eFigure 3. Histograms for the distribution of (A) mean sleep duration and (B) sleep duration SD

Supplement 2.
Data Sharing Statement
