# Supplementary material for: Social Determinants of Health and Long-Term, Device-Based Sleep Health
Source: JAMA Netw Open. 2026 Nov 10;9(11):e2643802. doi: 10.1001/jamanetworkopen.2026.43802 (PMC13752729; doi:10.1001/jamanetworkopen.2026.43802)
Supplement: Supplementary file 1 [file jamanetwopen-e2643802-s001.pdf]

Supplemental Online Content

Narla E, Zhu F, Beydoun MA, et al. Social determinants of health and long-term device-based sleep health. *JAMA Netw Open*. 2026;9(11):e2643802. doi:10.1001/jamanetworkopen.2026.43802

eTable 1. Assessment of social determinants of health (SDOH) in the All of Us Research Program
eTable 2. Comparison of key sociodemographic characteristics among the overall All of Us Research Program population (version 8), social determinants of health survey respondents, and the current study sample
eTable 3. Sensitivity analysis of associations between social determinants of health (SDOH) and the mean and SD of sleep duration among a subset of All of Us participants with depressive symptom assessments
eTable 4. Associations of social determinants of health with mean sleep duration and sleep duration SD stratified by age
eTable 5. Associations of social determinants of health with mean sleep duration and sleep duration SD stratified by sex
eTable 6. Associations of social determinants of health with mean sleep duration and sleep duration SD stratified by race
eTable 7. Associations of social determinants of health with mean sleep duration and sleep duration SD stratified by ethnicity
eFigure 1. Flowchart of the analytical sample
eFigure 2. Correlation between overall SDOH and SDOH domains
eFigure 3. Histograms for the distribution of (A) mean sleep duration and (B) sleep duration SD

This supplemental material has been provided by the authors to give readers additional information about their work.

© 2026 Narla E et al. *JAMA Network Open*.

eTable 1. **Assessment of Social Determinants of Health (SDOH) in the All of Us Research Program**

SDOH Measures	AoU Database (concept ids)[1]	Questions Used	Response Coding	Summary Score	Score Range	Missing Data Handling	Validity & Reliability
**Social/Community Context**							
**Social cohesion among neighbors**	40192463, 40192411, 40192499, 40192417	Social cohesion neighborhood scale (4-item)	1 = strongly agree…5 = strongly disagree	The scoring is the mean	1-5; lower = more socially cohesive	flagged as missing if any of the four items were missing a valid response	Cronbach's Alpha of 0.74, and a test-retest reliability of 0.65. Validation was done using census tract data.1
**Social support**	40192442, 40192480, 40192388, 40192511, 40192439, 40192528, 40192399, 40192446	RAND MOS social support survey (8-item)	1 = None of the time…5 = All of the time	The scoring is the mean	1-5; higher = more social support	It can be computed if 6 or more items have valid responses.	total score has a Cronbach's Alpha of 0.93.
**Loneliness**	40192507, 40192397, 40192504, 40192398, 40192501, 40192516, 40192390, 40192466	UCLA loneliness scale (8-item)	1 = Never…4 = Often	The scoring is the mean	1-4; lower= less lonely	It can be computed if 6 or more items have valid responses.	Cronbach's Alpha of 0.84 was reported for the 8-item scale

© 2026 Narla E et al. *JAMA Network Open*.

**Perceived discrimination**	40192466, 40192489, 40192416, 40192490, 40192380, 40192395, 40192496, 40192519, 40192451, 40192428	Everyday discrimination scale (9-item), plus attribution (1-item)	0=Never… 5=Almost every day	The scoring is the mean	1-5; lower = less discrimination	The scale is flagged as missing if more than two items do not have a valid score.	The coefficient Alpha was 0.88 in the original study.
**Perceived stress**	40192452, 40192381, 40192491, 40192419, 40192525, 40192506, 40192449, 40192445, 40192396, 40192462	Cohen's perceived stress scale (10-item)	1=Never…5= Very Often	The scoring is the mean	1-5; lower= lower perceived stress	can be scored if more than 8 items have a valid response.	The validated 10-item scale had a Cronbach's Alpha of 0.78.
**Daily spiritual experiences**	40192498, 40192475, 40192401, 40192443, 40192471, 40192415	Fetzer multidimensional measure of religiousness/spirituality (6-item)	1=Never or almost never…6=Many times a day (also includes 0=I do not believe in God or 0= I am not religious)	The scoring is the mean	1-6; higher = more spiritual	is flagged as missing if more than two items do not have a valid response.	Cronbach's Alpha for the original scale is 0.91

© 2026 Narla E et al. *JAMA Network Open*.

**Religious service attendance**	40192470	Religious service attendance nurses' health study (1-item)	1=Never… 5=More than once a week (no scoring if 0=I am not religious is chosen)	Scoring is the mean	1-5; higher= more attendance	If any item is missing	Data on reliability or validity were not available at the time of measure selection.
**English proficiency**	40192526	California health interview survey language spoken at home (1-item)		Single item			
**Economic Stability**							
**Food insecurity**	40192517, 40192426	Children's HealthWatch Hunger vital sign (2-sign)	True, sometimes true, or never true	Considered food insecure if any item is rated as sometimes true or often true	Considered foor insecure if any item is rated as sometimes true or often true	If any item is missing	Convergent validity was demonstrated using logistic regression models of health outcomes.

© 2026 Narla E et al. *JAMA Network Open*.

**Housing quality**	40192402	Accountable health communities (1-item)	1 = Bug infestation; 2 = Mold; 3 = Lead paint or pipes; 4 = Inadequate heat; 5 = Oven or stove not working; 6 = No or not working smoke detectors; 7 = Water leaks; 8 = None of the above	In this report, it is scored as a binary variable and set to zero if option 8 is the only item selected, and to 1 if one or more of options 1-7 are selected.	1= one or more issues	Just one question	Data on reliability or validity were not available at the time of measure selection.
**Neighborhood Environment**							
**Neighborhood physical disorder**	40192420, 40192522, 40192412, 40192469, 40192456, 40192386	Ross-Mirowsky perceived neighborhood disorder scale (6-item physical disorder subscale)	1=strongly disagree…4= strongly agree	The scoring is the mean	higher scores= more disorder	The scale is set to missing if more than one item does not have a valid response.	Coefficient alpha was 0.92 for the combined physical and social neighborhood disorder measure.

© 2026 Narla E et al. *JAMA Network Open*.

**Neighborhood social disorder**	40192500, 40192493, 40192457, 40192476, 40192404, 40192400, 40192384	Ross-Mirowsky perceived neighborhood disorder scale (7-item social disorder subscale)	1=strongly disagree…4= strongly agree	The scoring is the mean	higher scores= more disorder	The scale is set to missing if more than one item does not have a valid response.	Coefficient alpha was 0.92 for the combined physical and social neighborhood disorder measure.
**Neighborhood walkability**	40192436, 40192440, 40192437, 40192431, 40192410	Physical activity neighborhood environment scale (PANES) (5 core items)	1= strongly disagree…4= strongly agree	The scoring is the mean	higher scores = more walkable	flagged the score as missing if more than one item does not have a valid response.	good internal consistency reliability in All of Us (Cronbach's alpha =0.78) and may allow the ability to retain observations in rural geographies.
**Neighborhood crime**	40192492, 40192414	Physical activity neighborhood environment scale (PANES) crime (2-item subscale)	1=strongly disagree…4= strongly agree	The scoring is the mean	higher scores=less neighborhood safety	If either item is not a valid response (including Skip/Don't know), we flagged the scale as missing	Test-retest correlations were reported for individual items and showed acceptable reliability

© 2026 Narla E et al. *JAMA Network Open*.

**Neighborhood residential density**	40192458	Physical activity neighborhood environment scale (PANES) neighborhood housing type (1-item indicator)	Original response options (5 categories): 1-Detached single-family housing 2-Townhouses, row houses, apartments, or condos of 2–3 stories 3-Mix of single-family residences and townhouses, row houses, apartments or condos 4-Apartments or condos of 4–12 stories 5-Apartments or condos of more than 12 stories Coding is usually ordinal, 1–5, with higher scores reflecting greater perceived	Single item	Higher scores = greater residential density	If the response is Skip/Don’t know/missing, the density variable is coded as missing.	Test-retest reliability is acceptable (Sallis et al., 2010 reported ICCs in the 0.6–0.8 range for density and land-use items).

© 2026 Narla E et al. *JAMA Network Open*.

			residential density.				
**Healthcare Access and Quality**							
**Perceived discrimination in medical care settings**	40192497, 40192425, 40192503, 40192505, 40192423, 40192383, 40192394	Everyday discrimination scale in medical settings (7-item)	1=Never…5= Always	The scoring is the mean	higher scores= higher perceived discrimination in medical settings	the scale is flagged as missing if more than two items do not have valid responses	Cronbach's Alpha was 0.89, and the test-retest reliability was 0.58.

[1]All of Us concept IDs are unique numerical identifiers assigned to survey questions in the All of Us Research Program Database.

© 2026 Narla E et al. *JAMA Network Open*.

eTable 2. Comparison of key sociodemographic characteristics among the overall All of Us Research Program population (Version 8), social determinants of health survey respondents, and the current study sample

	All of Us Research Program population, Version 8 (n=633546)	Social determinants of health survey respondents (n=259107)	Analytical sample of the current study (n=12403)
Age at enrollment, mean (SD), y	51.0 (17.1)	53.9 (16.7)	53.2 (15.2)
Female, No. (%)	402336 (63.5)	171581 (66.2)	8719 (70.3)
Non-white,[1] No. (%)	249561 (39.4)	63289 (24.4)	1457 (11.7)
Hispanic, No. (%)	112751 (17.8)	30734 (11.9)	756 (6.1)
Education, No. (%)			
High school of less	174099 (27.5)	38902 (15.0)	747 (6.0)
Some college	167813 (26.5)	66243 (25.6)	2604 (21.0)
College graduate	149753 (23.6)	73456 (28.3)	4128 (33.3)
Advanced degree	141881 (22.4)	80506 (31.1)	4924 (39.7)
Annual household income, No. (%)			
<35K	191510 (30.2)	56047 (21.6)	1348 (10.9)
35-75K	125826 (19.9)	60480 (23.3)	2948 (23.8)
75-100K	56598 (8.9)	30366 (11.7)	1877 (15.1)
100-150K	69008 (10.9)	39556 (15.3)	2711 (21.9)
150-200K	32734 (5.2)	19094 (7.4)	1286 (10.4)
>200K	43165 (6.8)	25198 (9.7)	1596 (12.9)
missing	114705 (18.1)	28366 (10.9)	637 (5.1)
Employment status, No. (%)			
Employed	319503 (50.4)	137969 (53.2)	7987 (64.4)
Not employed[2]	167350 (26.4)	49528 (19.1)	1417 (11.4)
Retired	146693 (23.2)	71610 (27.6)	2999 (24.2)

[1]Non-White includes American Indian or Alaska Native, Asian, Black/African American/African, Middle Eastern or North African, Native Hawaiian or Other Pacific Islander, and more than one racial group.

[2]Includes individuals who were unemployed, homemakers, students, or unable to work

© 2026 Narla E et al. *JAMA Network Open*.

eTable 3. Sensitivity analysis of associations between social determinants of health (SDOH) and the mean and standard deviation of sleep duration among a subset of All of Us participants with depressive symptom assessments

		Mean sleep duration		Sleep duration SD	
	n	Model 3[1]	Sensitivity analysis[2]	Model 3[1]	Sensitivity analysis[2]
Overall SDOH score		beta (95% CI)	beta (95% CI)	beta (95% CI)	beta (95% CI)
T1	1590	reference	reference	reference	reference
T2	1610	4.14 (1.08, 7.21)	3.82 (0.71, 6.94)	-2.13 (-3.52, -0.74)	-0.36 (-1.74., 1.03)
T3	1473	8.33 (5.12,11.50)	7.79 (4.44, 11.10)	-4.72 (-6.18, -3.26)	-1.72 (-3.20, -0.23)
per 1-unit	4673	3.16 (1.81, 4.51)	2.90 (1.47, 4.33)	-2.45 (-3.06, -1.83)	-1.03 (-1.66, -0.40)
p-trend3		P < .001	P < .001	P < .001	P = .001

[1]Model 3 adjusted for age, sex, race, ethnicity, education, income, employment status, smoking and alcohol use

[2]Model 3 + depressive symptoms as measured by PHQ-9

[3]P-values for linear trends were calculated by modeling the SDOH score as a continuous variable

eTable 4. **Associations of Social Determinants of Health with Mean Sleep Duration and Sleep Duration SD stratified by Age**

	Mean sleep duration		Sleep duration SD	
**Age Group**	**beta (95% CI)[2]**	**P-Int.[1]**	**beta (95% CI)[2]**	**P-Int.[1]**
**Overall SDOH**				
<60	2.46 (1.45, 3.46)	0.05	-2.36 (-2.85, -1.88)	0.356
>=60	3.54 (2.18, 4.90)		-2.49 (-3.07, -1.90)	
**Social / Community Context**				
<60	0.02 (-1.03, 1.06)	0.01	-1.73 (-2.24, -1.22)	0.588
>=60	1.81 (0.60, 3.02)		-1.78 (-2.30, -1.26)	
**Economic Stability**				
<60	1.80 (0.87, 2.72)	0.072	-1.29 (-1.74, -0.85)	0.783
>=60	3.02 (1.62, 4.43)		-1.52 (-2.12, -0.91)	
**Neighborhood Environment**				
<60	1.37 (0.37, 2.37)	0.881	-0.78 (-1.26, -0.29)	0.782
>=60	1.16 (-0.06, 2.38)		-1.23 (-1.76, -0.70)	
**Health & Healthcare**				
<60	2.05 (1.07, 3.04)	0.902	-1.80 (-2.28, -1.33)	0.004
>=60	1.74 (0.48, 2.99)		-1.04 (-1.58, -0.50)	

[1] P-values for interactions were calculated by the Wald test of the cross-product terms.

[2] Values are β coefficients (95% CI), with SDOH variables modeled as continuous standardized scores. All estimates were adjusted for age, sex, race, ethnicity, education, income, employment status, smoking, and alcohol use.

© 2026 Narla E et al. *JAMA Network Open*.

eTable 5. **Associations of Social Determinants of Health with Mean Sleep Duration and Sleep Duration SD stratified by sex**

	Mean sleep duration		Sleep duration SD	
**Sex**	**beta (95% CI)[2.]**	**P-Int.[1]**	**beta (95% CI)[2]**	**P-Int.[1]**
**Overall SDOH**				
Male	0.70 (-0.98, 2.37)	0.1751	-2.87 (-3.59, -2.14)	0.6814
Female	2.38 (1.44, 3.33)		-2.66 (-3.09, -2.23)	
**Social / Community Context**				
Male	-0.10 (-1.58, 1.39)	0.7983	-2.12 (-2.76, -1.48)	0.8121
Female	0.32 (-0.63, 1.26)		-2.11 (-2.54, -1.68)	
**Economic Stability**				
Male	0.49 (-1.17, 2.14)	0.1665	-1.51 (-2.23, -0.79)	0.9641
Female	2.00 (1.09, 2.91)		-1.52 (-1.93, -1.10)	
**Neighborhood Environment**				
Male	0.16 (-1.34, 1.66)	0.4939	-1.40 (-2.06, -0.75)	0.4127
Female	1.04 (0.12, 1.97)		-1.00 (-1.42, -0.57)	
**Health & Healthcare**				
Male	0.83 (-0.69, 2.35)	0.1445	-1.20 (-1.86, -0.53)	0.2438
Female	1.89 (0.96, 2.83)		-1.73 (-2.16, -1.31)	

[1] P-values for interactions were calculated by the Wald test of the cross-product terms.

[2] Values are β coefficients (95% CI), with SDOH variables modeled as continuous standardized scores. All estimates were adjusted for age, sex, race, ethnicity, education, income, employment status, smoking, and alcohol use.

© 2026 Narla E et al. *JAMA Network Open*.

eTable 6. **Associations of Social Determinants of Health with Mean Sleep Duration and Sleep Duration SD stratified by race**

	Mean sleep duration		Sleep duration SD	
**Race**	**beta (95% CI)[2.]**	**P-Int.[1]**	**beta (95% CI)[2]**	**P-Int.[1]**
**Overall SDOH**				
White	1.88 (0.98, 2.77)	0.794	-2.77 (-3.17, -2.36)	0.562
Non-White	1.77 (-0.36, 3.90)		-2.46 (-3.39, -1.52)	
**Social / Community Context**				
White	0.07 (-0.79, 0.92)	0.65	-2.13 (-2.52, -1.75)	0.731
Non-White	0.81 (-1.42, 3.05)		-2.01 (-2.99, -1.03)	
**Economic Stability**				
White	1.80 (0.94, 2.67)	0.137	-1.60 (-1.99, -1.20)	0.345
Non-White	0.32 (-1.72, 2.36)		-1.12 (-2.02, -0.22)	
**Neighborhood Environment**				
White	0.48 (-0.37, 1.33)	0.042	-1.09 (-1.48, -0.71)	0.836
Non-White	2.37 (0.30, 4.44)		-1.26 (-2.18, -0.35)	
**Health & Healthcare**				
White	1.51 (0.66, 2.36)	0.935	-1.53 (-1.91, -1.14)	0.307
Non-White	1.52 (-0.68, 3.72)		-2.00 (-2.97, -1.03)	

[1] P-values for interactions were calculated by the Wald test of the cross-product terms.

[2] Values are β coefficients (95% CI), with SDOH variables modeled as continuous standardized scores. All estimates were adjusted for age, sex, race, ethnicity, education, income, employment status, smoking, and alcohol use.

© 2026 Narla E et al. *JAMA Network Open*.

eTable 7. **Associations of Social Determinants of Health with Mean Sleep Duration and Sleep Duration SD stratified by ethnicity**

	Mean sleep duration		Sleep duration SD	
**Ethnicity**	**beta (95% CI)[2]**	**P-Int.[1]**	**beta (95% CI)[2]**	**P-Int.[1]**
**Overall SDOH**				
Hispanic	3.96 (0.89, 7.02)	0.6383	-2.10 (-3.41, -0.78)	0.7828
Not Hispanic	2.93 (2.08, 3.79)		-2.66 (-3.04, -2.27)	
**Social / Community Context**				
Hispanic	3.39 (0.19, 6.58)	0.1356	-1.20 (-2.58, 0.17)	0.4373
Not Hispanic	1.26 (0.43, 2.09)		-2.05 (-2.42, -1.67)	
**Economic Stability**				
Hispanic	1.77 (-1.01, 4.56)	0.5361	-0.96 (-2.15, 0.24)	0.54
Not Hispanic	2.24 (1.41, 3.07)		-1.49 (-1.86, -1.11)	
**Neighborhood Environment**				
Hispanic	2.47 (-0.32, 5.26)	0.354	-1.64 (-2.83, -0.44)	0.2581
Not Hispanic	1.02 (0.20, 1.83)		-1.02 (-1.39, -0.64)	
**Health & Healthcare**				
Hispanic	2.21 (-1.00, 5.42)	0.8453	-1.35 (-2.72, 0.03)	0.751
Not Hispanic	1.86 (1.05, 2.68)		-1.54 (-1.91, -1.17)	

[1] P-values for interactions were calculated by the Wald test of the cross-product terms.

[2] Values are β coefficients (95% CI), with SDOH variables modeled as continuous standardized scores. All estimates were adjusted for age, sex, race, ethnicity, education, income, employment status, smoking, and alcohol use.

© 2026 Narla E et al. *JAMA Network Open*.

eFigure 1. **Flowchart of the analytical sample**

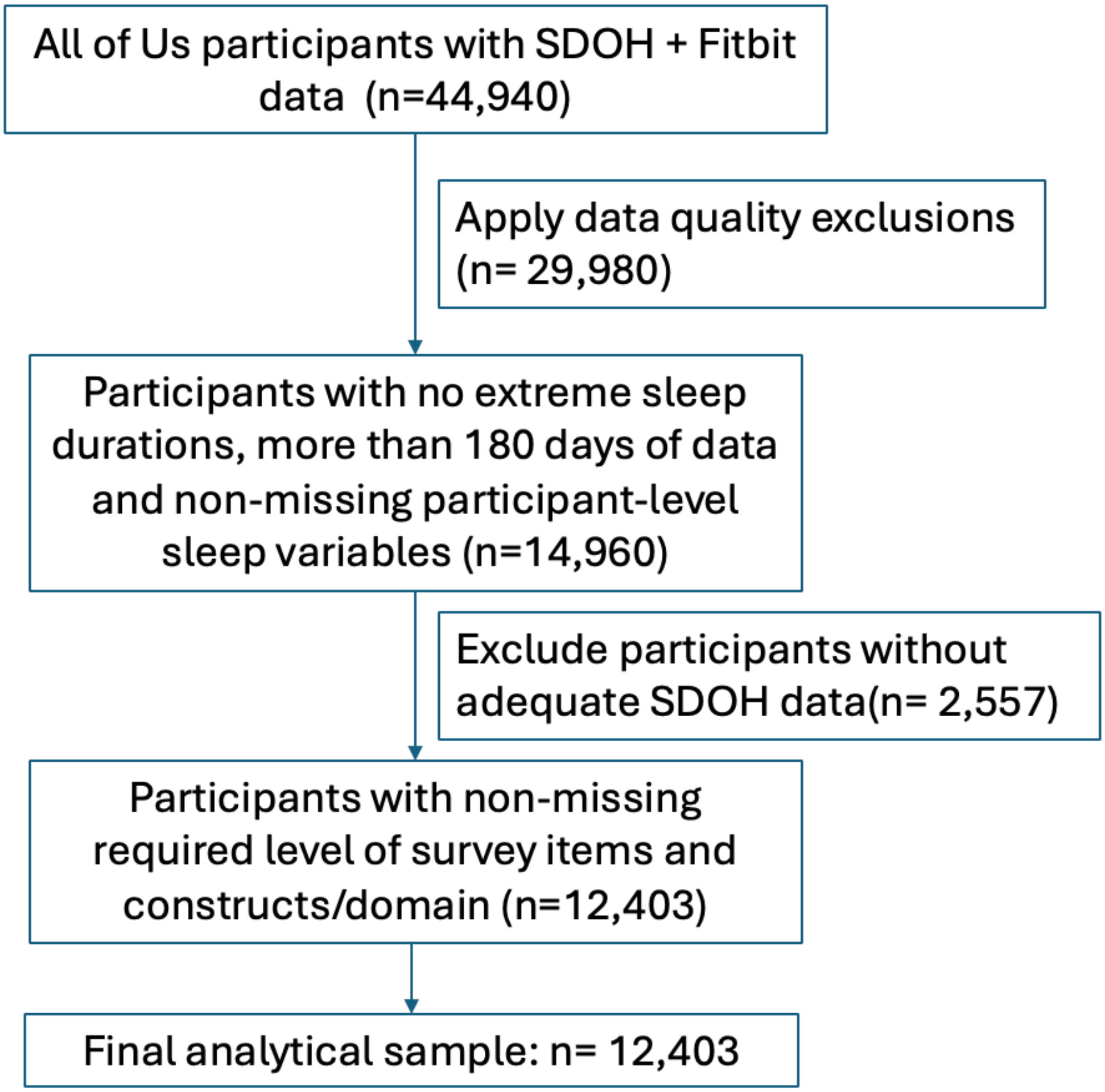

© 2026 Narla E et al. *JAMA Network Open*.

eFigure 2. **Correlation Between Overall SDOH and SDOH Domains**

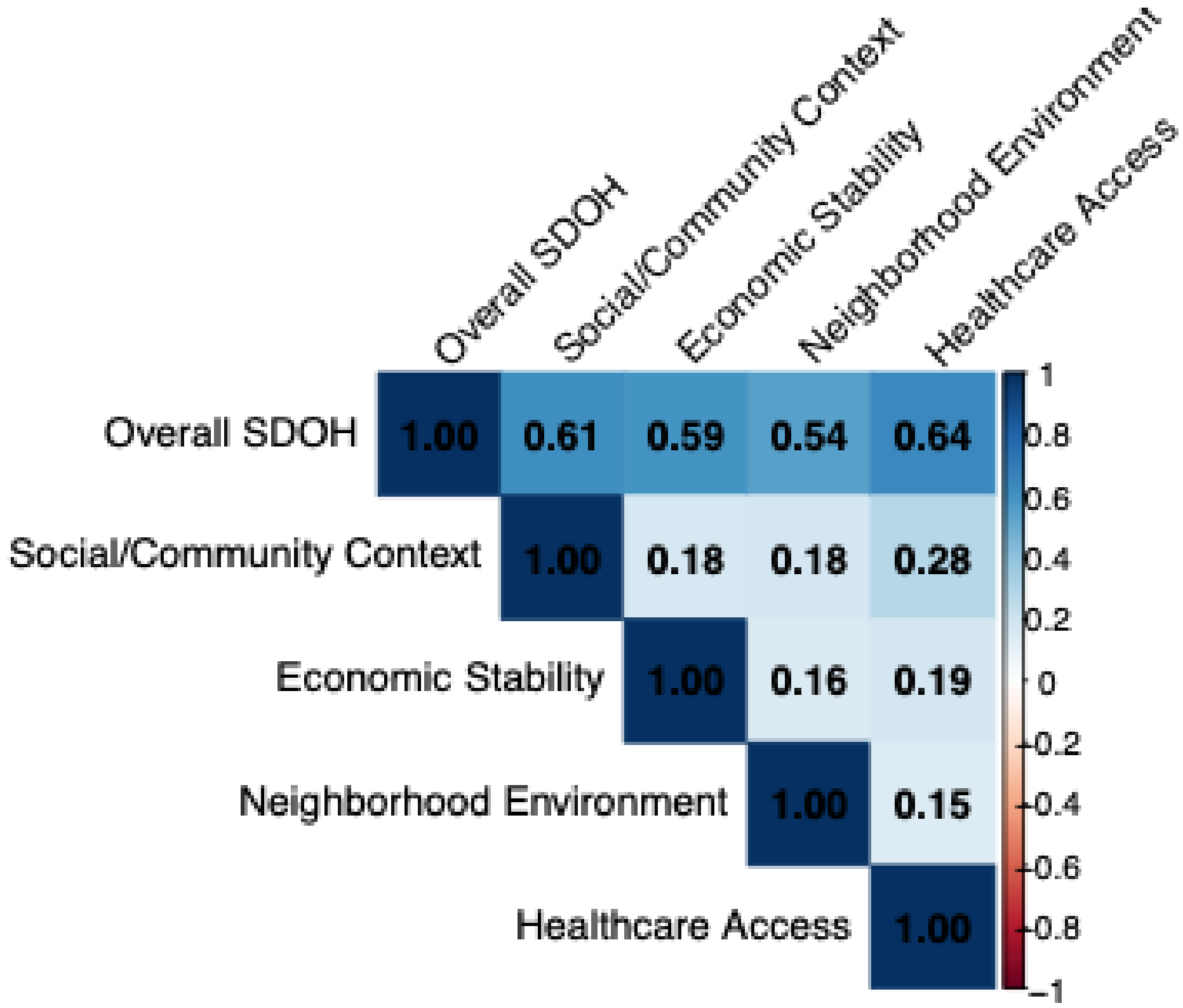

© 2026 Narla E et al. *JAMA Network Open*.

eFigure 3. **Histograms for the distribution of (A) mean sleep duration and (B) sleep duration SD**

A.

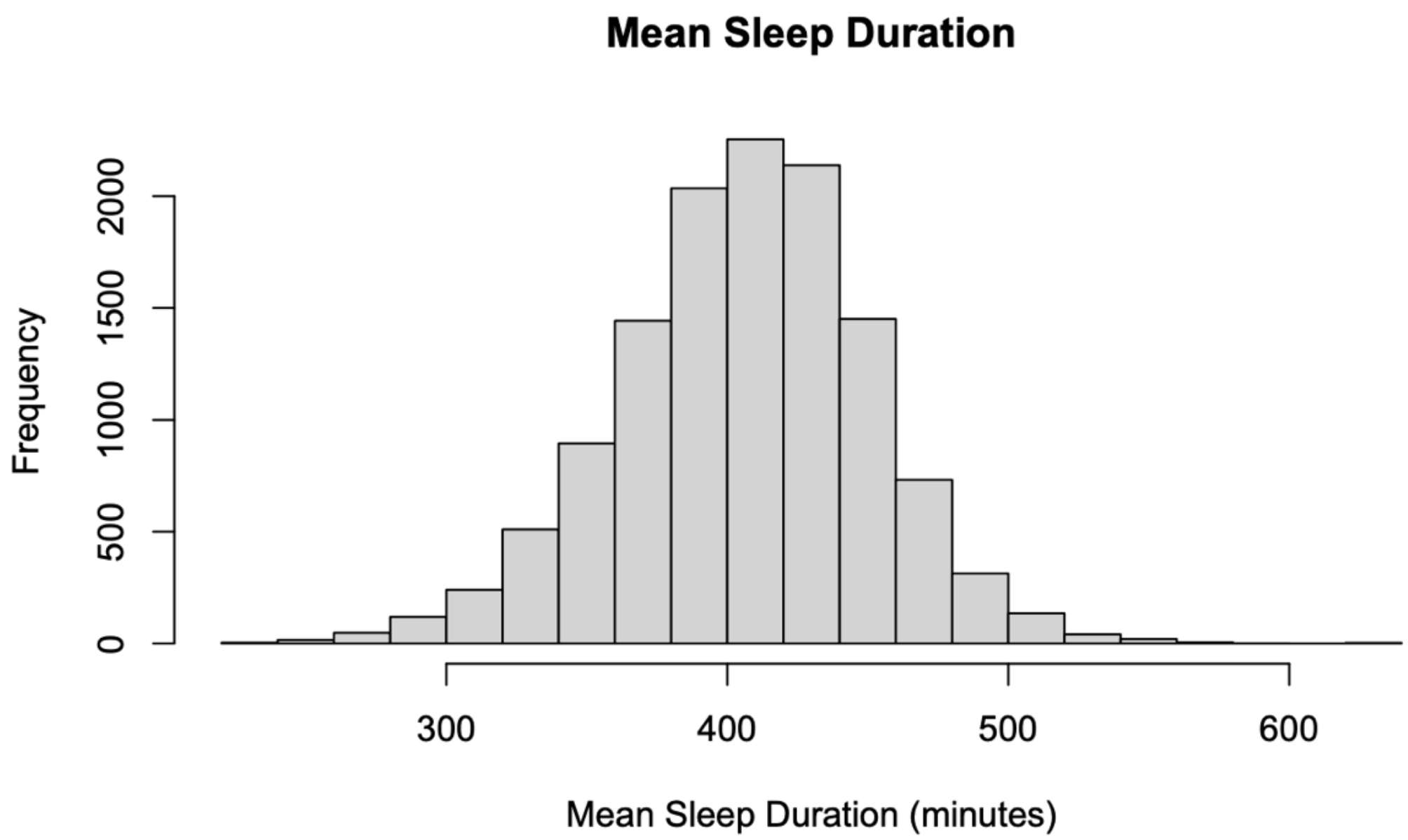

B.

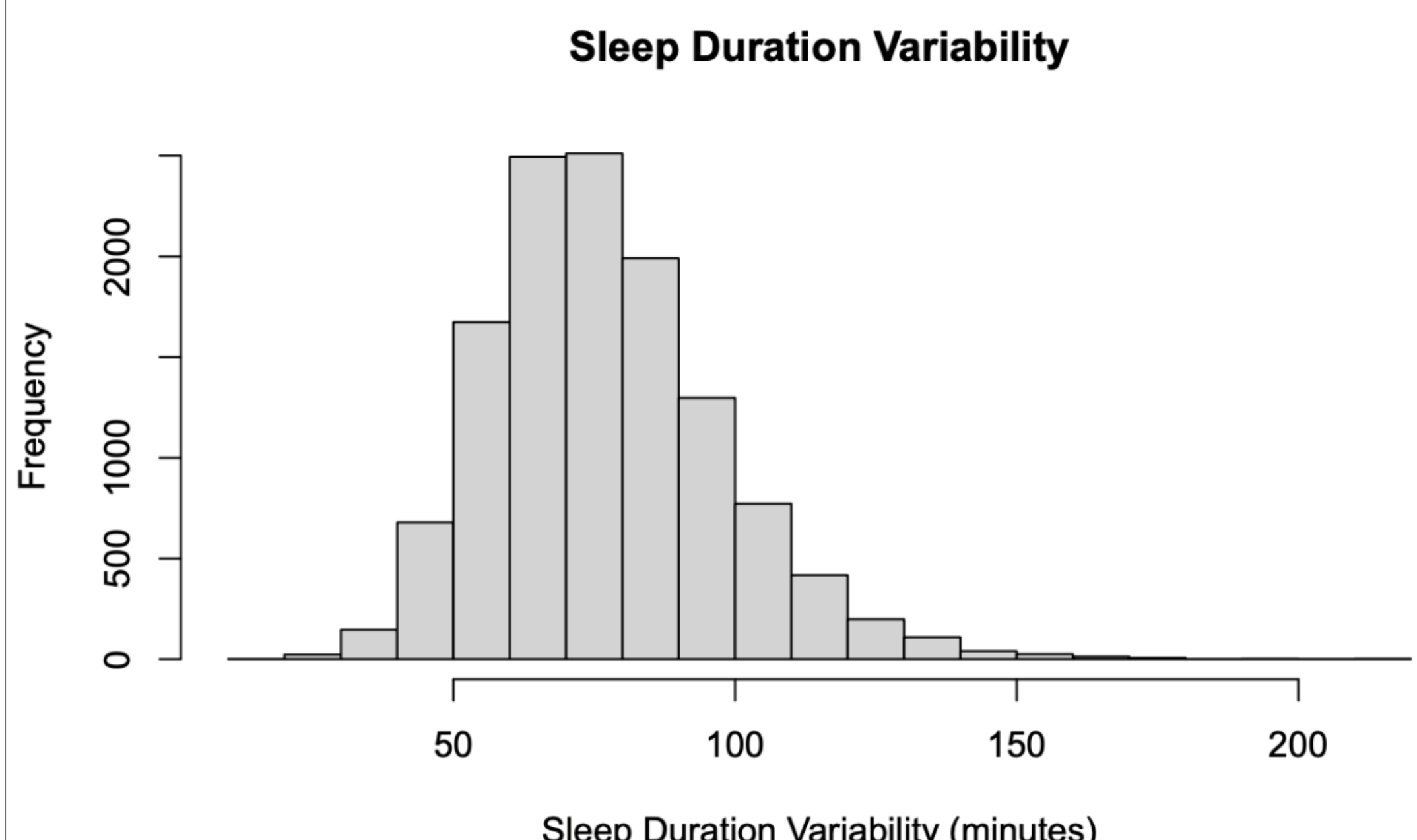

© 2026 Narla E et al. *JAMA Network Open*.
