# Supplementary material for: Social Determinants of Health and Long-Term, Device-Based Sleep Health
Source: JAMA Netw Open. 2026 Nov 10;9(11):e2643802. doi: 10.1001/jamanetworkopen.2026.43802 (PMC13752729; doi:10.1001/jamanetworkopen.2026.43802)
Supplement: Supplementary file 2 [file jamanetwopen-e2643802-s002.pdf]

Data Sharing Statement

Narla E. Social Determinants of Health and Long-Term, Device-Based Sleep Health. *JAMA Netw Open*. Published online November 9, 2026. doi:10.1001/jamanetworkopen.2026.43802

Data
Data available: No

Additional Information
Explanation for why data not available: This study used data from the NIH All of Us Research Program Controlled Tier Curated Data Repository, version 8, available to authorized researchers through the Researcher Workbench. Participant-level data cannot be shared publicly under All of Us data-use and participant-privacy policies. Supporting documentation (e.g., statistical/analytic code) is available upon request.
